# 16α-Bromo-17β-hydr­oxy-5α-androstan-3β-yl acetate

**DOI:** 10.1107/S1600536810007555

**Published:** 2010-03-06

**Authors:** Rui Shi, Chun-Sheng Zhang, Kun Wei

**Affiliations:** a Key Laboratory of Forest Resources Conservation and Use in the Southwest Mountains of China (Ministry of Education), Southwest Forestry University, Kunming 650224, People’s Republic of China; b School of Chemical Science and Technology, Key Laboratory of Medicinal Chemistry for Natural Resources, (Ministry of Education), Yunnan University, Kunming 650091, People’s Republic of China

## Abstract

The title compound, C_21_H_33_BrO_3_, an inter­mediate in the synthesis of the neuromuscular blocking agent rocuronium bromide, contains two independent mol­ecules in the asymmetric unit, which have almost identical geometries: in both mol­ecules, the steroidal rings *A*, *B* and *C* have slightly flattened chair conformations and ring *D* assumes a half-chair conformation. In the crystal, O—H⋯O hydrogen bonds and weak C—H⋯O and C—H⋯Br inter­actions help to establish the packing.

## Related literature

For further information on rocuronium bromide, see: Tuba *et al.* (2002 ▶); Auer (2007 ▶). For the synthesis, see: Fajkos & Sanda (1962 ▶).
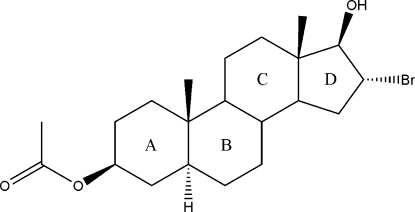


## Experimental

### 

#### Crystal data

C_21_H_33_BrO_3_
*M* *_r_* = 413.38
Monoclinic, 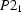

*a* = 11.1010 (12) Å
*b* = 7.6637 (8) Å
*c* = 24.383 (3) Å
β = 93.036 (2)°
*V* = 2071.5 (4) Å^3^
*Z* = 4
Mo *K*α radiation
μ = 2.00 mm^−1^
*T* = 298 K
0.26 × 0.18 × 0.12 mm

#### Data collection

Bruker SMART APEXII CCD diffractometer
Absorption correction: multi-scan (*SADABS*; Bruker, 2004 ▶) *T*_min_ = 0.624, *T*_max_ = 0.795
13607 measured reflections
9114 independent reflections
3737 reflections with *I* > 2σ(*I*)
*R*_int_ = 0.042

#### Refinement

*R*[*F* ^2^ > 2σ(*F* ^2^)] = 0.053
*wR*(*F* ^2^) = 0.125
*S* = 0.87
9114 reflections
459 parameters
1 restraint
H-atom parameters constrained
Δρ_max_ = 0.20 e Å^−3^
Δρ_min_ = −0.29 e Å^−3^
Absolute structure: Flack (1983 ▶), 3537 Friedel pairs
Flack parameter: 0.016 (9)

### 

Data collection: *APEX2* (Bruker, 2004 ▶); cell refinement: *SAINT* (Bruker, 2004 ▶); data reduction: *SAINT*; program(s) used to solve structure: *SHELXS97* (Sheldrick, 2008 ▶); program(s) used to refine structure: *SHELXL97* (Sheldrick, 2008 ▶); molecular graphics: *SHELXTL* (Sheldrick, 2008 ▶); software used to prepare material for publication: *SHELXTL*.

## Supplementary Material

Crystal structure: contains datablocks I, global. DOI: 10.1107/S1600536810007555/hb5324sup1.cif

Structure factors: contains datablocks I. DOI: 10.1107/S1600536810007555/hb5324Isup2.hkl

Additional supplementary materials:  crystallographic information; 3D view; checkCIF report

## Figures and Tables

**Table 1:** Hydrogen-bond geometry (Å, °)

*D*—H⋯*A*	*D*—H	H⋯*A*	*D*⋯*A*	*D*—H⋯*A*
O3—H3*A*⋯O6^i^	0.82	2.12	2.936 (6)	179
O6—H6⋯O5^ii^	0.82	1.97	2.757 (6)	160
C16—H16⋯O6^i^	0.98	2.56	3.347 (7)	137
C23—H22*A*⋯Br1^iii^	0.97	2.92	3.868 (6)	166
C37—H37⋯Br1^iv^	0.98	2.87	3.788 (6)	156

Symmetry codes: (i) 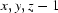; (ii) 
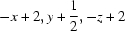
; (iii) 
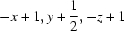
; (iv) 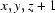.

**Fig. 1.**
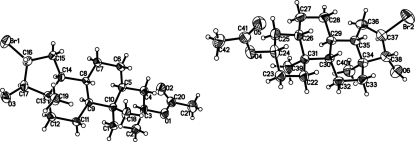
The molecular structure of (I) showing 30% probability displacement ellipsoids.

## supplementary crystallographic information

### Comment

As part of our ongoing investigation of the synthese of rocuronium bromide which is a new neuromuscular blocking agent (Tuba *et al.*, 2002; Auer, 2007), the title compound was obtained as an intermediate.

The title compound, C_21_H_33_BrO_3_, crystallizes in a monoclinic unit cell. The molecular structure is shown in Fig. 1., which contains two crystallographically independent molecules in the asymmetric unit which have almost identical geometry. Rings *A*, *B* and *C* have chair conformations which are slightly flattened and *D* ring assumes a half-chair conformation in both molecules.

Cohesion of the crystals can be attributed to van der Waals and weak molecular O—H···O interactions (Table 1).

### Experimental

The title compound was synthesized according to the procedure of Fajkos *et al.* (1962).  Colourless prisms of (I) were obtained from a mixture of ethyl acetate and petroleum ether by slow evaporation at room temperature.

### Refinement

Methyl H atoms were placed in calculated positions with C—H = 0.96Å and the torsion angle was refined to fit the electron density; *U*_iso_(H) = 1.5*U*_eq_(C). Other H atoms were placed in calculated positions with C—H = 0.97–0.98 Å and O—H = 0.82 Å, and refined in riding mode; *U*_iso_(H) = 1.2*U*_eq_(C, O).

### Crystal data

**Table:**

C_21_H_33_BrO_3_	*F*(000) = 872
*M**_r_* = 413.38	*D*_x_ = 1.326 Mg m^−^^3^
Monoclinic, *P*2_1_	Mo *K*α radiation, λ = 0.71073 Å
Hall symbol: P 2yb	Cell parameters from 9114 reflections
*a* = 11.1010 (12) Å	θ = 2.2–28.4°
*b* = 7.6637 (8) Å	µ = 2.00 mm^−^^1^
*c* = 24.383 (3) Å	*T* = 298 K
β = 93.036 (2)°	Prism, colourless
*V* = 2071.5 (4) Å^3^	0.26 × 0.18 × 0.12 mm
*Z* = 4	

### Data collection

**Table:**

Bruker SMART CCD diffractometer	9114 independent reflections
Radiation source: fine-focus sealed tube	3737 reflections with *I* > 2σ(*I*)
graphite	*R*_int_ = 0.042
ω scans	θ_max_ = 28.4°, θ_min_ = 1.7°
Absorption correction: multi-scan (*SADABS*; Bruker, 2004)	*h* = −14→14
*T*_min_ = 0.624, *T*_max_ = 0.795	*k* = −10→9
13607 measured reflections	*l* = −32→21

### Refinement

**Table:**

Refinement on *F*^2^	Hydrogen site location: inferred from neighbouring sites
Least-squares matrix: full	H-atom parameters constrained
*R*[*F*^2^ > 2σ(*F*^2^)] = 0.053	*w* = 1/[σ^2^(*F*_o_^2^)] where *P* = (*F*_o_^2^ + 2*F*_c_^2^)/3
*wR*(*F*^2^) = 0.125	(Δ/σ)_max_ = 0.001
*S* = 0.87	Δρ_max_ = 0.20 e Å^−^^3^
9114 reflections	Δρ_min_ = −0.29 e Å^−^^3^
459 parameters	Extinction correction: *SHELXL97* (Sheldrick, 2008), Fc^*^=kFc[1+0.001xFc^2^λ^3^/sin(2θ)]^-1/4^
1 restraint	Extinction coefficient: 0.0010 (4)
Primary atom site location: structure-invariant direct methods	Absolute structure: Flack (1983), 3537 Friedel pairs
Secondary atom site location: difference Fourier map	Flack parameter: 0.016 (9)

### Special details

**Table:**

Geometry. All e.s.d.'s (except the e.s.d. in the dihedral angle between two l.s. planes)are estimated using the full covariance matrix. The cell e.s.d.'s are takeninto account individually in the estimation of e.s.d.'s in distances, anglesand torsion angles; correlations between e.s.d.'s in cell parameters are onlyused when they are defined by crystal symmetry. An approximate (isotropic)treatment of cell e.s.d.'s is used for estimating e.s.d.'s involving l.s. planes.
Refinement. Refinement of *F*^2^ against ALL reflections. The weighted *R*-factor *wR* and goodness of fit *S* are based on *F*^2^, conventional *R*-factors *R* are based on *F*, with *F* set to zero for negative *F*^2^. The threshold expression of *F*^2^ > σ(*F*^2^) is used only for calculating *R*-factors(gt) *etc*, and is not relevant to the choice of reflections for refinement. *R*-factors based on *F*^2^ are statistically about twice as large as those based on *F*, and *R*-factors based on ALL data will be even larger.

### Fractional atomic coordinates and isotropic or equivalent isotropic displacement parameters (Å^2^)

**Table:**

	*x*	*y*	*z*	*U*_iso_*/*U*_eq_	
Br1	0.33539 (6)	0.31032 (9)	0.21950 (3)	0.0820 (2)	
Br2	0.69555 (9)	0.09933 (13)	1.21764 (4)	0.1268 (4)	
C1	0.1761 (6)	0.9883 (7)	0.4929 (2)	0.0587 (17)	
H1A	0.1025	0.9662	0.4708	0.070*	
H1B	0.2129	1.0927	0.4788	0.070*	
C2	0.1441 (6)	1.0208 (8)	0.5527 (2)	0.0650 (17)	
H2A	0.2162	1.0542	0.5744	0.078*	
H2B	0.0868	1.1161	0.5539	0.078*	
C3	0.0910 (5)	0.8609 (7)	0.5769 (2)	0.0515 (15)	
H3	0.0135	0.8363	0.5573	0.062*	
C4	0.1719 (5)	0.7031 (7)	0.5725 (2)	0.0540 (15)	
H4A	0.1311	0.6009	0.5859	0.065*	
H4B	0.2452	0.7204	0.5953	0.065*	
C5	0.2041 (5)	0.6725 (7)	0.5133 (2)	0.0476 (14)	
H5	0.1271	0.6549	0.4925	0.057*	
C6	0.2747 (5)	0.5055 (7)	0.5065 (2)	0.0573 (16)	
H6A	0.2322	0.4093	0.5227	0.069*	
H6B	0.3531	0.5162	0.5257	0.069*	
C7	0.2912 (5)	0.4670 (7)	0.4461 (2)	0.0537 (15)	
H7A	0.3437	0.3665	0.4434	0.064*	
H7B	0.2136	0.4372	0.4285	0.064*	
C8	0.3445 (4)	0.6188 (6)	0.41582 (19)	0.0386 (12)	
H8	0.4282	0.6340	0.4299	0.046*	
C9	0.2764 (4)	0.7906 (7)	0.42545 (18)	0.0409 (12)	
H9	0.1943	0.7722	0.4098	0.049*	
C10	0.2628 (4)	0.8334 (7)	0.48690 (19)	0.0414 (12)	
C11	0.3289 (5)	0.9417 (7)	0.3922 (2)	0.0535 (15)	
H11A	0.2796	1.0448	0.3964	0.064*	
H11B	0.4096	0.9680	0.4072	0.064*	
C12	0.3347 (5)	0.8993 (7)	0.3304 (2)	0.0591 (16)	
H12A	0.2533	0.8894	0.3141	0.071*	
H12B	0.3744	0.9943	0.3123	0.071*	
C13	0.4018 (4)	0.7322 (7)	0.3212 (2)	0.0449 (14)	
C14	0.3454 (4)	0.5866 (7)	0.35444 (19)	0.0452 (13)	
H14	0.2602	0.5843	0.3417	0.054*	
C15	0.3986 (5)	0.4182 (7)	0.3326 (2)	0.0562 (15)	
H15A	0.3421	0.3223	0.3347	0.067*	
H15B	0.4730	0.3877	0.3531	0.067*	
C16	0.4224 (5)	0.4631 (7)	0.2720 (2)	0.0572 (16)	
H16	0.5091	0.4535	0.2667	0.069*	
C17	0.3837 (5)	0.6541 (7)	0.2639 (2)	0.0550 (15)	
H17	0.2967	0.6537	0.2546	0.066*	
C18	0.3861 (4)	0.8748 (8)	0.5150 (2)	0.0655 (18)	
H18A	0.3770	0.8956	0.5534	0.098*	
H18B	0.4190	0.9769	0.4986	0.098*	
H18C	0.4395	0.7779	0.5107	0.098*	
C19	0.5391 (5)	0.7546 (8)	0.3358 (2)	0.0682 (18)	
H19A	0.5794	0.6453	0.3310	0.102*	
H19B	0.5510	0.7918	0.3733	0.102*	
H19C	0.5716	0.8406	0.3120	0.102*	
C20	−0.0136 (6)	0.8036 (11)	0.6582 (3)	0.0664 (16)	
C22	0.9194 (5)	0.5784 (8)	0.9099 (2)	0.0636 (16)	
H21A	0.8961	0.6953	0.9203	0.076*	
H21B	0.9936	0.5498	0.9307	0.076*	
C23	0.9434 (5)	0.5775 (9)	0.8484 (2)	0.0681 (17)	
H22A	0.8720	0.6170	0.8273	0.082*	
H22B	1.0090	0.6568	0.8415	0.082*	
C24	0.9754 (5)	0.3973 (9)	0.8309 (2)	0.0665 (18)	
H23	1.0531	0.3638	0.8488	0.080*	
C25	0.8804 (5)	0.2637 (8)	0.8443 (2)	0.0741 (19)	
H24A	0.9075	0.1481	0.8344	0.089*	
H24B	0.8061	0.2884	0.8231	0.089*	
C26	0.8571 (5)	0.2682 (7)	0.9057 (2)	0.0562 (16)	
H25	0.9342	0.2405	0.9252	0.067*	
C27	0.7673 (5)	0.1253 (8)	0.9218 (3)	0.0680 (17)	
H26A	0.6883	0.1490	0.9045	0.082*	
H26B	0.7940	0.0126	0.9091	0.082*	
C28	0.7594 (5)	0.1218 (8)	0.9839 (2)	0.0645 (17)	
H27A	0.8366	0.0850	1.0006	0.077*	
H27B	0.6995	0.0365	0.9934	0.077*	
C29	0.7262 (4)	0.2992 (7)	1.0077 (2)	0.0488 (13)	
H28	0.6447	0.3299	0.9934	0.059*	
C30	0.8140 (5)	0.4417 (7)	0.9899 (2)	0.0491 (15)	
H29	0.8943	0.4047	1.0040	0.059*	
C31	0.8203 (5)	0.4497 (7)	0.9258 (2)	0.0486 (15)	
C32	0.7916 (5)	0.6187 (7)	1.0166 (2)	0.0653 (16)	
H31A	0.7153	0.6652	1.0019	0.078*	
H31B	0.8547	0.6992	1.0072	0.078*	
C33	0.7886 (5)	0.6073 (8)	1.0793 (2)	0.0627 (16)	
H32A	0.8679	0.5751	1.0946	0.075*	
H32B	0.7683	0.7207	1.0939	0.075*	
C34	0.6968 (5)	0.4737 (7)	1.0961 (3)	0.0523 (16)	
C35	0.7276 (4)	0.2988 (8)	1.0701 (2)	0.0525 (14)	
H34	0.8115	0.2754	1.0823	0.063*	
C36	0.6519 (6)	0.1651 (8)	1.1007 (3)	0.075 (2)	
H35A	0.5739	0.1470	1.0817	0.090*	
H35B	0.6935	0.0540	1.1043	0.090*	
C37	0.6380 (5)	0.2497 (8)	1.1572 (3)	0.072 (2)	
H37	0.5523	0.2739	1.1613	0.087*	
C38	0.7055 (5)	0.4229 (8)	1.1569 (3)	0.0611 (17)	
H38D	0.7903	0.4024	1.1683	0.073*	
C39	0.6978 (5)	0.5097 (9)	0.8988 (2)	0.073 (2)	
H38A	0.6996	0.4990	0.8596	0.109*	
H38B	0.6835	0.6293	0.9082	0.109*	
H38C	0.6343	0.4381	0.9117	0.109*	
C41	1.0671 (6)	0.3018 (11)	0.7497 (3)	0.0749 (18)	
C42	1.0744 (6)	0.3298 (10)	0.6886 (2)	0.100 (2)	
H41A	1.1387	0.2604	0.6754	0.150*	
H41B	1.0898	0.4508	0.6816	0.150*	
H41C	0.9995	0.2961	0.6702	0.150*	
C21	−0.0195 (6)	0.8451 (10)	0.7174 (2)	0.092 (2)	
H42A	−0.0679	0.9476	0.7216	0.138*	
H42B	0.0605	0.8658	0.7330	0.138*	
H42C	−0.0548	0.7489	0.7360	0.138*	
O1	0.0693 (3)	0.8992 (5)	0.63361 (16)	0.0658 (11)	
O2	−0.0737 (4)	0.6942 (7)	0.63403 (18)	0.0890 (15)	
O3	0.4395 (4)	0.7394 (5)	0.22015 (17)	0.0806 (13)	
H3A	0.5005	0.6859	0.2128	0.121*	
O4	0.9865 (3)	0.4031 (6)	0.77130 (16)	0.0752 (13)	
O5	1.1284 (4)	0.2026 (7)	0.77638 (19)	0.0934 (15)	
O6	0.6557 (4)	0.5418 (6)	1.19406 (19)	0.0867 (15)	
H6	0.7092	0.6055	1.2072	0.130*	
C40	0.5684 (5)	0.5365 (9)	1.0800 (3)	0.083 (2)	
H43A	0.5521	0.6417	1.0997	0.124*	
H43B	0.5115	0.4482	1.0891	0.124*	
H43C	0.5613	0.5590	1.0413	0.124*	

### Atomic displacement parameters (Å^2^)

**Table:**

	*U*^11^	*U*^22^	*U*^33^	*U*^12^	*U*^13^	*U*^23^
Br1	0.0874 (5)	0.0966 (5)	0.0636 (4)	−0.0233 (5)	0.0199 (3)	−0.0292 (4)
Br2	0.1275 (7)	0.1339 (8)	0.1199 (8)	−0.0042 (6)	0.0157 (6)	0.0642 (6)
C1	0.077 (4)	0.046 (4)	0.055 (4)	0.001 (3)	0.016 (3)	−0.001 (3)
C2	0.079 (4)	0.059 (4)	0.059 (4)	0.011 (3)	0.021 (3)	−0.007 (3)
C3	0.059 (4)	0.060 (4)	0.036 (3)	−0.003 (3)	0.012 (3)	−0.005 (3)
C4	0.057 (4)	0.058 (4)	0.047 (4)	−0.004 (3)	0.010 (3)	0.002 (3)
C5	0.055 (3)	0.046 (4)	0.043 (4)	0.002 (3)	0.008 (3)	−0.001 (3)
C6	0.078 (4)	0.047 (4)	0.048 (4)	−0.002 (3)	0.012 (3)	0.008 (3)
C7	0.073 (4)	0.047 (4)	0.042 (4)	0.003 (3)	0.015 (3)	0.006 (3)
C8	0.044 (3)	0.036 (3)	0.036 (3)	0.001 (3)	0.002 (2)	0.007 (2)
C9	0.046 (3)	0.040 (3)	0.037 (3)	−0.004 (3)	0.001 (2)	0.000 (3)
C10	0.043 (3)	0.042 (3)	0.040 (3)	−0.001 (3)	0.009 (2)	−0.003 (3)
C11	0.074 (4)	0.042 (4)	0.047 (4)	0.008 (3)	0.022 (3)	0.003 (3)
C12	0.082 (4)	0.049 (4)	0.048 (4)	0.005 (3)	0.019 (3)	0.007 (3)
C13	0.043 (3)	0.049 (3)	0.044 (4)	0.000 (3)	0.008 (3)	−0.001 (3)
C14	0.053 (3)	0.051 (3)	0.031 (3)	−0.003 (3)	0.004 (3)	−0.001 (3)
C15	0.084 (4)	0.045 (3)	0.041 (4)	0.002 (3)	0.013 (3)	−0.007 (3)
C16	0.063 (4)	0.056 (4)	0.053 (4)	−0.004 (3)	0.008 (3)	−0.008 (3)
C17	0.058 (4)	0.071 (5)	0.036 (3)	0.007 (3)	0.007 (3)	0.000 (3)
C18	0.060 (4)	0.090 (5)	0.046 (4)	−0.023 (3)	0.006 (3)	−0.012 (3)
C19	0.068 (4)	0.080 (5)	0.058 (4)	−0.023 (3)	0.017 (3)	−0.001 (3)
C20	0.070 (4)	0.076 (5)	0.055 (4)	−0.001 (5)	0.018 (4)	0.012 (4)
C22	0.079 (4)	0.066 (4)	0.047 (4)	−0.010 (4)	0.008 (3)	0.002 (3)
C23	0.076 (4)	0.070 (5)	0.058 (4)	−0.009 (4)	0.009 (3)	0.005 (4)
C24	0.061 (4)	0.093 (5)	0.046 (4)	0.006 (4)	0.002 (3)	0.006 (4)
C25	0.080 (4)	0.080 (5)	0.061 (4)	0.006 (4)	−0.009 (4)	−0.007 (3)
C26	0.054 (3)	0.064 (5)	0.049 (4)	0.000 (3)	−0.006 (3)	−0.005 (3)
C27	0.079 (4)	0.049 (4)	0.076 (5)	−0.010 (4)	0.002 (4)	−0.011 (3)
C28	0.068 (4)	0.056 (4)	0.070 (5)	−0.012 (3)	0.009 (3)	−0.005 (3)
C29	0.039 (3)	0.042 (3)	0.065 (4)	−0.001 (3)	−0.002 (2)	0.005 (3)
C30	0.043 (3)	0.045 (4)	0.059 (4)	0.000 (3)	0.000 (3)	−0.004 (3)
C31	0.044 (3)	0.049 (4)	0.052 (4)	−0.005 (3)	−0.007 (3)	0.001 (3)
C32	0.088 (4)	0.046 (4)	0.063 (4)	−0.012 (3)	0.016 (3)	−0.001 (3)
C33	0.082 (4)	0.054 (4)	0.054 (4)	−0.010 (4)	0.015 (3)	−0.006 (3)
C34	0.040 (3)	0.053 (4)	0.065 (4)	0.004 (3)	0.010 (3)	0.002 (3)
C35	0.042 (3)	0.052 (4)	0.064 (4)	0.007 (3)	0.009 (3)	0.004 (4)
C36	0.073 (4)	0.062 (4)	0.092 (6)	−0.015 (4)	0.017 (4)	0.011 (4)
C37	0.053 (4)	0.089 (6)	0.077 (5)	0.007 (4)	0.015 (3)	0.017 (4)
C38	0.042 (3)	0.070 (4)	0.072 (5)	−0.004 (3)	0.014 (3)	0.007 (4)
C39	0.064 (4)	0.095 (5)	0.058 (4)	0.017 (4)	−0.009 (3)	0.006 (4)
C41	0.091 (5)	0.069 (5)	0.065 (5)	0.024 (5)	0.014 (4)	−0.006 (4)
C42	0.144 (6)	0.097 (6)	0.061 (4)	0.005 (5)	0.034 (4)	−0.018 (4)
C21	0.109 (5)	0.109 (6)	0.061 (5)	−0.002 (5)	0.036 (4)	0.004 (4)
O1	0.081 (3)	0.073 (3)	0.046 (3)	−0.011 (2)	0.025 (2)	−0.007 (2)
O2	0.081 (3)	0.118 (4)	0.068 (3)	−0.027 (3)	0.014 (3)	−0.001 (3)
O3	0.110 (4)	0.080 (3)	0.056 (3)	0.007 (2)	0.040 (3)	0.012 (2)
O4	0.077 (3)	0.097 (3)	0.051 (3)	0.024 (3)	0.000 (2)	0.005 (2)
O5	0.097 (4)	0.096 (4)	0.088 (4)	0.031 (3)	0.009 (3)	0.002 (3)
O6	0.078 (3)	0.106 (4)	0.079 (3)	−0.012 (3)	0.032 (3)	−0.021 (3)
C40	0.064 (4)	0.091 (5)	0.094 (5)	0.020 (4)	0.007 (4)	−0.002 (4)

### Geometric parameters (Å, °)

**Table:**

Br1—C16	1.952 (5)	C22—H21B	0.9700
Br2—C37	1.951 (6)	C23—C24	1.494 (8)
C1—C10	1.540 (7)	C23—H22A	0.9700
C1—C2	1.540 (7)	C23—H22B	0.9700
C1—H1A	0.9700	C24—O4	1.466 (6)
C1—H1B	0.9700	C24—C25	1.518 (8)
C2—C3	1.495 (7)	C24—H23	0.9800
C2—H2A	0.9700	C25—C26	1.533 (7)
C2—H2B	0.9700	C25—H24A	0.9700
C3—O1	1.446 (6)	C25—H24B	0.9700
C3—C4	1.514 (7)	C26—C31	1.537 (7)
C3—H3	0.9800	C26—C27	1.546 (7)
C4—C5	1.523 (7)	C26—H25	0.9800
C4—H4A	0.9700	C27—C28	1.523 (7)
C4—H4B	0.9700	C27—H26A	0.9700
C5—C6	1.514 (7)	C27—H26B	0.9700
C5—C10	1.551 (7)	C28—C29	1.530 (8)
C5—H5	0.9800	C28—H27A	0.9700
C6—C7	1.522 (7)	C28—H27B	0.9700
C6—H6A	0.9700	C29—C35	1.521 (6)
C6—H6B	0.9700	C29—C30	1.542 (7)
C7—C8	1.515 (7)	C29—H28	0.9800
C7—H7A	0.9700	C30—C32	1.531 (8)
C7—H7B	0.9700	C30—C31	1.569 (7)
C8—C14	1.517 (6)	C30—H29	0.9800
C8—C9	1.542 (6)	C31—C39	1.549 (7)
C8—H8	0.9800	C32—C33	1.532 (7)
C9—C11	1.545 (7)	C32—H31A	0.9700
C9—C10	1.549 (6)	C32—H31B	0.9700
C9—H9	0.9800	C33—C34	1.517 (7)
C10—C18	1.531 (6)	C33—H32A	0.9700
C11—C12	1.546 (7)	C33—H32B	0.9700
C11—H11A	0.9700	C34—C35	1.528 (7)
C11—H11B	0.9700	C34—C38	1.532 (8)
C12—C13	1.504 (7)	C34—C40	1.536 (7)
C12—H12A	0.9700	C35—C36	1.542 (7)
C12—H12B	0.9700	C35—H34	0.9800
C13—C17	1.525 (7)	C36—C37	1.538 (8)
C13—C14	1.532 (7)	C36—H35A	0.9700
C13—C19	1.556 (7)	C36—H35B	0.9700
C14—C15	1.526 (7)	C37—C38	1.524 (8)
C14—H14	0.9800	C37—H37	0.9800
C15—C16	1.555 (7)	C38—O6	1.417 (7)
C15—H15A	0.9700	C38—H38D	0.9800
C15—H15B	0.9700	C39—H38A	0.9600
C16—C17	1.536 (7)	C39—H38B	0.9600
C16—H16	0.9800	C39—H38C	0.9600
C17—O3	1.420 (6)	C41—O5	1.191 (7)
C17—H17	0.9800	C41—O4	1.316 (7)
C18—H18A	0.9600	C41—C42	1.509 (8)
C18—H18B	0.9600	C42—H41A	0.9600
C18—H18C	0.9600	C42—H41B	0.9600
C19—H19A	0.9600	C42—H41C	0.9600
C19—H19B	0.9600	C21—H42A	0.9600
C19—H19C	0.9600	C21—H42B	0.9600
C20—O2	1.207 (7)	C21—H42C	0.9600
C20—O1	1.343 (7)	O3—H3A	0.8200
C20—C21	1.482 (8)	O6—H6	0.8200
C22—C23	1.537 (7)	C40—H43A	0.9600
C22—C31	1.541 (7)	C40—H43B	0.9600
C22—H21A	0.9700	C40—H43C	0.9600
			
C10—C1—C2	113.1 (4)	C22—C23—H22A	109.7
C10—C1—H1A	109.0	C24—C23—H22B	109.7
C2—C1—H1A	109.0	C22—C23—H22B	109.7
C10—C1—H1B	109.0	H22A—C23—H22B	108.2
C2—C1—H1B	109.0	O4—C24—C23	106.8 (5)
H1A—C1—H1B	107.8	O4—C24—C25	109.2 (5)
C3—C2—C1	110.9 (5)	C23—C24—C25	112.5 (5)
C3—C2—H2A	109.5	O4—C24—H23	109.4
C1—C2—H2A	109.5	C23—C24—H23	109.4
C3—C2—H2B	109.5	C25—C24—H23	109.4
C1—C2—H2B	109.5	C24—C25—C26	110.5 (5)
H2A—C2—H2B	108.1	C24—C25—H24A	109.5
O1—C3—C2	107.5 (4)	C26—C25—H24A	109.5
O1—C3—C4	111.1 (4)	C24—C25—H24B	109.5
C2—C3—C4	112.3 (4)	C26—C25—H24B	109.5
O1—C3—H3	108.6	H24A—C25—H24B	108.1
C2—C3—H3	108.6	C25—C26—C31	113.1 (5)
C4—C3—H3	108.6	C25—C26—C27	112.1 (5)
C3—C4—C5	111.2 (5)	C31—C26—C27	112.0 (5)
C3—C4—H4A	109.4	C25—C26—H25	106.3
C5—C4—H4A	109.4	C31—C26—H25	106.3
C3—C4—H4B	109.4	C27—C26—H25	106.3
C5—C4—H4B	109.4	C28—C27—C26	109.7 (5)
H4A—C4—H4B	108.0	C28—C27—H26A	109.7
C6—C5—C4	112.5 (4)	C26—C27—H26A	109.7
C6—C5—C10	113.3 (4)	C28—C27—H26B	109.7
C4—C5—C10	113.3 (4)	C26—C27—H26B	109.7
C6—C5—H5	105.6	H26A—C27—H26B	108.2
C4—C5—H5	105.6	C27—C28—C29	112.9 (5)
C10—C5—H5	105.6	C27—C28—H27A	109.0
C5—C6—C7	111.1 (4)	C29—C28—H27A	109.0
C5—C6—H6A	109.4	C27—C28—H27B	109.0
C7—C6—H6A	109.4	C29—C28—H27B	109.0
C5—C6—H6B	109.4	H27A—C28—H27B	107.8
C7—C6—H6B	109.4	C35—C29—C28	112.7 (5)
H6A—C6—H6B	108.0	C35—C29—C30	108.1 (4)
C8—C7—C6	113.1 (4)	C28—C29—C30	110.7 (4)
C8—C7—H7A	109.0	C35—C29—H28	108.4
C6—C7—H7A	109.0	C28—C29—H28	108.4
C8—C7—H7B	109.0	C30—C29—H28	108.4
C6—C7—H7B	109.0	C32—C30—C29	112.8 (5)
H7A—C7—H7B	107.8	C32—C30—C31	114.0 (5)
C7—C8—C14	112.3 (4)	C29—C30—C31	111.7 (4)
C7—C8—C9	112.0 (4)	C32—C30—H29	105.8
C14—C8—C9	108.5 (4)	C29—C30—H29	105.8
C7—C8—H8	108.0	C31—C30—H29	105.8
C14—C8—H8	108.0	C26—C31—C22	107.1 (4)
C9—C8—H8	108.0	C26—C31—C39	112.1 (5)
C8—C9—C11	111.0 (4)	C22—C31—C39	109.0 (5)
C8—C9—C10	113.7 (4)	C26—C31—C30	108.0 (4)
C11—C9—C10	114.0 (4)	C22—C31—C30	110.2 (4)
C8—C9—H9	105.8	C39—C31—C30	110.5 (5)
C11—C9—H9	105.8	C30—C32—C33	112.7 (5)
C10—C9—H9	105.8	C30—C32—H31A	109.0
C18—C10—C1	110.2 (5)	C33—C32—H31A	109.0
C18—C10—C9	110.3 (4)	C30—C32—H31B	109.0
C1—C10—C9	110.4 (4)	C33—C32—H31B	109.0
C18—C10—C5	111.4 (4)	H31A—C32—H31B	107.8
C1—C10—C5	107.1 (4)	C34—C33—C32	111.0 (5)
C9—C10—C5	107.3 (4)	C34—C33—H32A	109.4
C12—C11—C9	113.0 (4)	C32—C33—H32A	109.4
C12—C11—H11A	109.0	C34—C33—H32B	109.4
C9—C11—H11A	109.0	C32—C33—H32B	109.4
C12—C11—H11B	109.0	H32A—C33—H32B	108.0
C9—C11—H11B	109.0	C33—C34—C35	108.2 (4)
H11A—C11—H11B	107.8	C33—C34—C38	115.1 (5)
C13—C12—C11	111.7 (5)	C35—C34—C38	100.1 (5)
C13—C12—H12A	109.3	C33—C34—C40	110.3 (5)
C11—C12—H12A	109.3	C35—C34—C40	113.2 (5)
C13—C12—H12B	109.3	C38—C34—C40	109.6 (5)
C11—C12—H12B	109.3	C29—C35—C34	115.0 (5)
H12A—C12—H12B	107.9	C29—C35—C36	120.6 (5)
C12—C13—C17	115.4 (5)	C34—C35—C36	104.1 (4)
C12—C13—C14	108.8 (4)	C29—C35—H34	105.3
C17—C13—C14	99.3 (4)	C34—C35—H34	105.3
C12—C13—C19	111.0 (5)	C36—C35—H34	105.3
C17—C13—C19	109.4 (4)	C37—C36—C35	103.7 (5)
C14—C13—C19	112.5 (4)	C37—C36—H35A	111.0
C8—C14—C15	120.3 (4)	C35—C36—H35A	111.0
C8—C14—C13	115.3 (4)	C37—C36—H35B	111.0
C15—C14—C13	104.8 (4)	C35—C36—H35B	111.0
C8—C14—H14	105.0	H35A—C36—H35B	109.0
C15—C14—H14	105.0	C38—C37—C36	106.9 (5)
C13—C14—H14	105.0	C38—C37—Br2	112.1 (4)
C14—C15—C16	103.5 (4)	C36—C37—Br2	112.5 (4)
C14—C15—H15A	111.1	C38—C37—H37	108.4
C16—C15—H15A	111.1	C36—C37—H37	108.4
C14—C15—H15B	111.1	Br2—C37—H37	108.4
C16—C15—H15B	111.1	O6—C38—C37	110.4 (5)
H15A—C15—H15B	109.0	O6—C38—C34	116.7 (5)
C17—C16—C15	105.8 (4)	C37—C38—C34	102.7 (5)
C17—C16—Br1	111.2 (4)	O6—C38—H38D	108.9
C15—C16—Br1	112.8 (4)	C37—C38—H38D	108.9
C17—C16—H16	109.0	C34—C38—H38D	108.9
C15—C16—H16	109.0	C31—C39—H38A	109.5
Br1—C16—H16	109.0	C31—C39—H38B	109.5
O3—C17—C13	117.8 (5)	H38A—C39—H38B	109.5
O3—C17—C16	113.9 (4)	C31—C39—H38C	109.5
C13—C17—C16	103.5 (4)	H38A—C39—H38C	109.5
O3—C17—H17	107.0	H38B—C39—H38C	109.5
C13—C17—H17	107.0	O5—C41—O4	122.6 (6)
C16—C17—H17	107.0	O5—C41—C42	124.8 (6)
C10—C18—H18A	109.5	O4—C41—C42	112.6 (7)
C10—C18—H18B	109.5	C41—C42—H41A	109.5
H18A—C18—H18B	109.5	C41—C42—H41B	109.5
C10—C18—H18C	109.5	H41A—C42—H41B	109.5
H18A—C18—H18C	109.5	C41—C42—H41C	109.5
H18B—C18—H18C	109.5	H41A—C42—H41C	109.5
C13—C19—H19A	109.5	H41B—C42—H41C	109.5
C13—C19—H19B	109.5	C20—C21—H42A	109.5
H19A—C19—H19B	109.5	C20—C21—H42B	109.5
C13—C19—H19C	109.5	H42A—C21—H42B	109.5
H19A—C19—H19C	109.5	C20—C21—H42C	109.5
H19B—C19—H19C	109.5	H42A—C21—H42C	109.5
O2—C20—O1	122.3 (6)	H42B—C21—H42C	109.5
O2—C20—C21	125.0 (6)	C20—O1—C3	118.0 (5)
O1—C20—C21	112.6 (7)	C17—O3—H3A	109.5
C23—C22—C31	113.9 (5)	C41—O4—C24	118.3 (5)
C23—C22—H21A	108.8	C38—O6—H6	109.5
C31—C22—H21A	108.8	C34—C40—H43A	109.5
C23—C22—H21B	108.8	C34—C40—H43B	109.5
C31—C22—H21B	108.8	H43A—C40—H43B	109.5
H21A—C22—H21B	107.7	C34—C40—H43C	109.5
C24—C23—C22	109.8 (5)	H43A—C40—H43C	109.5
C24—C23—H22A	109.7	H43B—C40—H43C	109.5
			
C10—C1—C2—C3	−56.7 (7)	C23—C24—C25—C26	−55.6 (7)
C1—C2—C3—O1	177.1 (4)	C24—C25—C26—C31	56.1 (6)
C1—C2—C3—C4	54.6 (7)	C24—C25—C26—C27	−176.0 (5)
O1—C3—C4—C5	−174.8 (4)	C25—C26—C27—C28	173.2 (5)
C2—C3—C4—C5	−54.5 (6)	C31—C26—C27—C28	−58.4 (6)
C3—C4—C5—C6	−174.3 (4)	C26—C27—C28—C29	55.4 (6)
C3—C4—C5—C10	55.6 (6)	C27—C28—C29—C35	−175.7 (4)
C4—C5—C6—C7	172.7 (5)	C27—C28—C29—C30	−54.5 (6)
C10—C5—C6—C7	−57.2 (6)	C35—C29—C30—C32	−51.0 (6)
C5—C6—C7—C8	52.8 (6)	C28—C29—C30—C32	−174.9 (5)
C6—C7—C8—C14	−172.6 (4)	C35—C29—C30—C31	179.0 (4)
C6—C7—C8—C9	−50.2 (6)	C28—C29—C30—C31	55.1 (6)
C7—C8—C9—C11	−177.6 (4)	C25—C26—C31—C22	−54.9 (6)
C14—C8—C9—C11	−53.1 (5)	C27—C26—C31—C22	177.2 (5)
C7—C8—C9—C10	52.3 (5)	C25—C26—C31—C39	64.6 (6)
C14—C8—C9—C10	176.7 (4)	C27—C26—C31—C39	−63.3 (6)
C2—C1—C10—C18	−66.3 (6)	C25—C26—C31—C30	−173.5 (4)
C2—C1—C10—C9	171.7 (5)	C27—C26—C31—C30	58.6 (6)
C2—C1—C10—C5	55.1 (6)	C23—C22—C31—C26	55.4 (6)
C8—C9—C10—C18	67.6 (5)	C23—C22—C31—C39	−66.0 (6)
C11—C9—C10—C18	−61.0 (6)	C23—C22—C31—C30	172.6 (5)
C8—C9—C10—C1	−170.4 (4)	C32—C30—C31—C26	173.7 (5)
C11—C9—C10—C1	61.0 (5)	C29—C30—C31—C26	−56.9 (5)
C8—C9—C10—C5	−53.9 (5)	C32—C30—C31—C22	57.1 (6)
C11—C9—C10—C5	177.5 (4)	C29—C30—C31—C22	−173.5 (4)
C6—C5—C10—C18	−63.9 (6)	C32—C30—C31—C39	−63.4 (6)
C4—C5—C10—C18	65.8 (6)	C29—C30—C31—C39	66.0 (6)
C6—C5—C10—C1	175.4 (5)	C29—C30—C32—C33	52.4 (6)
C4—C5—C10—C1	−54.9 (5)	C31—C30—C32—C33	−178.7 (4)
C6—C5—C10—C9	56.8 (5)	C30—C32—C33—C34	−54.9 (6)
C4—C5—C10—C9	−173.5 (4)	C32—C33—C34—C35	56.3 (6)
C8—C9—C11—C12	53.4 (6)	C32—C33—C34—C38	167.3 (5)
C10—C9—C11—C12	−176.7 (4)	C32—C33—C34—C40	−68.0 (6)
C9—C11—C12—C13	−54.2 (6)	C28—C29—C35—C34	179.1 (4)
C11—C12—C13—C17	164.2 (5)	C30—C29—C35—C34	56.4 (5)
C11—C12—C13—C14	53.7 (6)	C28—C29—C35—C36	−55.0 (6)
C11—C12—C13—C19	−70.6 (5)	C30—C29—C35—C36	−177.7 (5)
C7—C8—C14—C15	−51.1 (6)	C33—C34—C35—C29	−59.9 (6)
C9—C8—C14—C15	−175.4 (4)	C38—C34—C35—C29	179.3 (4)
C7—C8—C14—C13	−178.1 (4)	C40—C34—C35—C29	62.7 (6)
C9—C8—C14—C13	57.6 (5)	C33—C34—C35—C36	166.1 (5)
C12—C13—C14—C8	−58.1 (6)	C38—C34—C35—C36	45.3 (5)
C17—C13—C14—C8	−179.1 (4)	C40—C34—C35—C36	−71.3 (6)
C19—C13—C14—C8	65.3 (6)	C29—C35—C36—C37	−158.6 (5)
C12—C13—C14—C15	167.4 (5)	C34—C35—C36—C37	−27.8 (6)
C17—C13—C14—C15	46.4 (5)	C35—C36—C37—C38	−0.5 (6)
C19—C13—C14—C15	−69.2 (5)	C35—C36—C37—Br2	−124.0 (4)
C8—C14—C15—C16	−161.2 (4)	C36—C37—C38—O6	153.6 (5)
C13—C14—C15—C16	−29.5 (5)	Br2—C37—C38—O6	−82.7 (5)
C14—C15—C16—C17	1.1 (6)	C36—C37—C38—C34	28.5 (6)
C14—C15—C16—Br1	−120.7 (4)	Br2—C37—C38—C34	152.2 (4)
C12—C13—C17—O3	72.5 (6)	C33—C34—C38—O6	78.4 (6)
C14—C13—C17—O3	−171.5 (5)	C35—C34—C38—O6	−165.9 (4)
C19—C13—C17—O3	−53.6 (6)	C40—C34—C38—O6	−46.6 (7)
C12—C13—C17—C16	−160.8 (4)	C33—C34—C38—C37	−160.7 (5)
C14—C13—C17—C16	−44.8 (5)	C35—C34—C38—C37	−45.0 (5)
C19—C13—C17—C16	73.1 (5)	C40—C34—C38—C37	74.3 (6)
C15—C16—C17—O3	156.7 (5)	O2—C20—O1—C3	−3.7 (9)
Br1—C16—C17—O3	−80.4 (5)	C21—C20—O1—C3	174.9 (5)
C15—C16—C17—C13	27.6 (5)	C2—C3—O1—C20	157.4 (5)
Br1—C16—C17—C13	150.5 (3)	C4—C3—O1—C20	−79.5 (6)
C31—C22—C23—C24	−56.6 (7)	O5—C41—O4—C24	2.3 (10)
C22—C23—C24—O4	175.1 (4)	C42—C41—O4—C24	−176.1 (5)
C22—C23—C24—C25	55.2 (7)	C23—C24—O4—C41	146.1 (6)
O4—C24—C25—C26	−174.0 (5)	C25—C24—O4—C41	−91.9 (6)

### Hydrogen-bond geometry (Å, °)

**Table:**

*D*—H···*A*	*D*—H	H···*A*	*D*···*A*	*D*—H···*A*
O3—H3A···O6^i^	0.82	2.12	2.936 (6)	179
O6—H6···O5^ii^	0.82	1.97	2.757 (6)	160
C16—H16···O6^i^	0.98	2.56	3.347 (7)	137
C23—H22A···Br1^iii^	0.97	2.92	3.868 (6)	166
C37—H37···Br1^iv^	0.98	2.87	3.788 (6)	156

Symmetry codes: (i) *x*, *y*, *z*−1; (ii) −*x*+2, *y*+1/2, −*z*+2; (iii) −*x*+1, *y*+1/2, −*z*+1; (iv) *x*, *y*, *z*+1.
